# Supplementary material for: Who am I? Narratives as a window to transformative moments in critical care
Source: PLoS One. 2021 Nov 15;16(11):e0259976. doi: 10.1371/journal.pone.0259976 (PMC8592467; doi:10.1371/journal.pone.0259976)
Supplement: S1 Appendix — (DOCX) [file pone.0259976.s001.docx]

| **Journal** | **Website** |
| --- | --- |
| American Journal of Respiratory and Critical Care Medicine | <https://www.atsjournals.org/journal/ajrccm> |
| Intensive Care Medicine | <https://www.springer.com/journal/134> |
| Critical Care Medicine | <https://journals.lww.com/ccmjournal/pages/default.aspx> |
| Chest | <http://chestjournal.chestpubs.org/> |
| Critical Care | <https://ccforum.biomedcentral.com/> |
| Annals of Intensive Care | <https://annalsofintensivecare.springeropen.com/> |
| European Heart Journal: Acute Cardiovascular Care | <https://journals.sagepub.com/home/acc> |
| Journal of Trauma and Acute Care Surgery | <https://journals.lww.com/jtrauma/pages/default.aspx> |
| Current Opinion in Critical Care | <https://journals.lww.com/co-criticalcare/pages/default.aspx> |
| Pediatric Critical Care Medicine | <https://journals.lww.com/pccmjournal/pages/default.aspx> |
| Critical Care Clinics | <https://www.journals.elsevier.com/critical-care-clinics> |
| Scandinavian Journal of Trauma, Resuscitation, and Emergency Medicine | <https://sjtrem.biomedcentral.com/> |
| Journal of Critical Care | <https://www.journals.elsevier.com/journal-of-critical-care> |
| Journal of Intensive Care | <https://jintensivecare.biomedcentral.com/> |
| Shock | <https://journals.lww.com/shockjournal/pages/default.aspx> |
| Neurocritical Care | <https://www.springer.com/journal/12028> |
| Emergency Medicine Journal | <https://emj.bmj.com/> |
| Critical Care and Resuscitation | <https://ccr.cicm.org.au/> |
| Anaesthesia, critical care, & pain medicine | <https://www.journals.elsevier.com/anaesthesia-critical-care-and-pain-medicine> |
| Seminars in Respiratory and Critical Care Medicine | <https://www.thieme.com/books-main/internal-medicine/product/2166-seminars-in-respiratory-and-critical-care-medicine> |
| Respiratory Care | <http://rc.rcjournal.com/> |
| Archives of Trauma Research | <http://www.archtrauma.com/> |
| Heart and Lung: Journal of Acute and Critical Care | <https://www.journals.elsevier.com/heart-and-lung> |
| Journal of Intensive Care Medicine | <https://journals.sagepub.com/home/jic> |
| European Journal of Trauma and Emergency Medicine | <https://www.springer.com/journal/68> |
| Anaesthesia and Intensive Care | <https://journals.sagepub.com/home/aic> |
| Anaesthesiology Intensive Therapy | <https://www.termedia.pl/Journal/Anaesthesiology_Intensive_Therapy-118/Numer-2-2019> |
| Trauma Surgery and Acute Care Open | <https://tsaco.bmj.com/> |
| Journal of the Intensive Care Society | <https://journals.sagepub.com/home/inc> |
| Critical Care Research and Practice | <https://www.hindawi.com/journals/ccrp/> |
