# Supplementary material for: Who am I? Narratives as a window to transformative moments in critical care
Source: PLoS One. 2021 Nov 15;16(11):e0259976. doi: 10.1371/journal.pone.0259976 (PMC8592467; doi:10.1371/journal.pone.0259976)
Supplement: S2 Appendix — (Narratives were published in the section “From the Inside”, Intensive Care Medicine Journal). (DOCX) [file pone.0259976.s002.docx]

**Analyzes of narratives written by ICU clinicians**

**(Narratives were published in the section “From the Inside”, *Intensive Care Medicine Journal*)**

| **Publication Year** | **Author**  **Country**  **Characteristics** | ***Title*** | **Reflections & Quotes** | **Themes** |
| --- | --- | --- | --- | --- |
| 2021 | Kanaris  UK  Paediatric intensivist writing about working in an adult COVID ICU | *Moral distress in the intensive care unit during the pandemic: the burden of dying alone* | We look death in the eye, we play chess with him, and… roughly 9 out of 10 times, we win. That is our comfort zone.  Every death affects us, but we are proficient at facilitating a good death for those that we cannot save.  …what kept us awake at night during the pandemic was moral distress; the thought that somehow, we were complicit in allowing these patients to die alone  Morphine and midazolam are poor substitutes for a human, familial touch.  Compassion rounds had done their job… The next day, I wanted to be a doctor again. | **Tension:**   - Life vs Death: intensivists are in the business of thwarting death and providing dignity at the end of life for patients that we cannot save - Duty vs Humanity/Connection vs Isolation: contrast between the duty to maintain social isolation policy and the resultant deprivation of human contact for the patients - Resilience vs Moral Distress: commentary on the moral distress of witnessing what appears to be a “bad” or “unfair” death and how the institution of “compassion rounds” improved resilience in both patients and the medical care team |
| 2020 | Langlume  France  Four Intensivists reflection on language used to speak about patients | *Metaphor use in the ICU: rigor with words!* | They were deeply shocked by the use of the word “vegetable” to describe their beloved husband and father  One might draw a parallel between the use of metaphor, which allows simplification, rapidity, and efficient transfer of meaning, and the work of the intensivist, who requires exactly the same properties…  Which characteristics of a vegetable are highlighted and transposed to the patient, in the doctor’s mind? …this metaphor illustrates the inability of a comatose patient to think or speak… on a deeper level, it illustrates the dehumanization of the patient in the doctor’s mind | **Tension:**   - Dehumanization vs Humanity: commentary on how the use of words can betray the common reflex of dehumanizing patients in the critical care environment, special attention to the need to be aware of this reflex and work to prevent it - Art vs Science: contrasting the artistic use of metaphor in literature with the need for similar simplification and rapid information transfer in the critical care environment |
| 2020 | Curley  US  Clinician and researcher | *Alone, the hardest part* | …it behooves us to facilitate loving relationships and connections in our clinical worlds, as we continue to practice social distancing and isolation in our personal worlds. Dying alone, despite adhering to social distancing, should not be part of dying at all. | **Tension:**   - Connection vs Isolation: commentary regarding the tragedy of dying alone and unsupported and how it could possibly be fixed by facilitating family presence at bedside |
| 2020 | Lehr  Canada  Trainee, poem contrasting the mundane catastrophies with the tragedies seen in ICU | *Life or Linen* | What else is left to say? / It was their only kid. Fatality! / It was his favorite kit. Calamity! / So what now? /  We’ll have to cancel dinner plans.  A crisis in the eyes of the beholder… Beware of emotional numbness! | **Tension:**   - Extraordinary vs Ordinary: contrast of extraordinary ICU experiences with the experiences of other individuals outside of the ICU - Compassion vs Apathy: warning against dismissing “common” complaints just because they don’t always meet the intensity of the trauma faced in the ICU – maintaining compassion and recognition of the issues that other people face |
| 2020 | Vellelonga  Italy  Intensivists reflecting on patient care during the time of COVID-19 | *Doctor-Patient Relationship at the time of COVID-19: Travel Notes* | Introduce yourself. Explain who you are, your role and share your medical plan. Behind a mask, a gown and face-shield we are all the same in the eyes of a fearful patient. Make your connection personal.  Uncertainty is also a fundamental part of life and disease. With COVID-19 we know less but we do not care less. With uncertainty comes hope.  Acknowledge the fear of dying alone. In disease and death fear makes us all equal. We are all human and that is the only thing that matters. | **Tension**:   - Technology vs Humanity: science, technology, and lack of data as a barrier to human connection and compassion; use of technology to support human connection - Attachment vs Detachment: PPE as a barrier to human connection – inability to see facial expressions makes eye contact and direct, purposeful acknowledgement important in building connection - Hope vs despair: maintaining hope in spite of uncertainty, both with respect to data, therapy, and prognosis; providing dignity in the context of fear |
| 2020 | Mema  Canada  Intensivist, poem commemorating love and closeness with recently deceased partner | *Hands* | And then holding was hard because of the pain, and kissing was difficult because of the shortness of breath. We are left holding hands, and a love that was and is so immense | **Tension:**   - Attachment vs. detachment: contrast of physical and emotional closeness before and after the barrier created by illness – emphasis on how the connection is preserved through simple human contact (holding hands) even when other forms of affection were less possible |
| 2020 | Mema  Canada  Intensivist, reflection on dealing with personal loss while working in an environment that demands we witness the loss of others | *A Grief Unobserved* | For me, grief was lonliness. The person I was closest to and could talk to anything about was gone.  I returned to work and felt the looks. Most of my colleagues were unsure about how to act, afraid of making a mistake.  A family decides to pursue comfort care. This time I decide I cannot help. I cannot see agonal breathing after extubation. I cannot see a body getting bluer and cooler. It reminds me of the savage despair of sitting by the bed of my dying love.  My work reminds me of my loss, but also allows me to be among people whose suffering I now understand more deeply and whose pain I can help alleviate. | **Tension:**   - Connection vs Isolation: feeling alone despite being surrounded by well-meaning colleagues that didn’t know how to properly act; recognizing similar isolation in grieving families and trying to bridge create a connection through purposeful human interaction - Role as Physician vs Role as Bereaved Caregiver: contrast between feelings as bereaved caregiver and responsibilities as staff physician to provide care to the grieving families of critically ill children |
| 2020 | Einav  Israel  Intensivist using dictionary definitions to illustrate the progression of her feelings during COVID | *Inexorable* | Inexorable is the word that comes to mind as we watch the disease spread. Sending tentacles of fever and dry cough from country to country.  Hubris. Can we do more? Should we do more? Will we ask ourselves what more we could have done when the dust settles?  As the death toll of colloeagues in medicine rises and we face the inevitability of loss, it is no longer “Will this happen to me?”. It is “When will it happen?” and “Who will I lose?”.  …mostly we are concerned we will need to choose. If those who should determine policy look the other way, will it befall us to select who gets what?  This incredible local and global community of friends. Solidarity. And gratitude.  Endurance is the word that comes to mind as we accept what awaits us and hope for the best. | **Tension:**   - Hope vs Fear: maintaining hope and resilience in the context of uncertainty and fear, providing hope to others despite our fears that the illness will take our families - Connection vs Isolation: maintaining connection with our profession, solidarity with our colleagues as a way of promoting endurance through this period of physical and social isolation; inability to care for and maintain contact with our families due to fear of spreading illness - Choice vs Chance: feelings of helplessness as we are forced to make decisions to stem the tide of what seems like unstoppable illness – do our choices matter? Do they make a difference; what happens if we must decide who lives and dies? Is that best left to policy? To chance? - Individual vs Society: we are trained and programmed to focus on our individual patient but now are asked to make conflicting decisions for the greater good of society – how can we reconcile this with our beliefs or our training? |
| 2020 | Gusmao Florez  Brazil  Intensivist writing a poem about his dad’s final moments in ICU | *Forgive me for not letting you go* | Why open your chest to fix what we no longer could? Why pump on your heart when it no longer would? I wanted to contest death, and I believed I should  We cut the thread too late, forgive me. Our moment for farewell I failed to see  …the joy you gave my aching heart with each and every smile, are memories you left behind, and these will not depart | **Tension:**   - Life vs Death: living on after death through the memories of others; timing of death – “contesting death” through CPR, resuscitation, even though it may have been better to let death take its course |
| 2019 | Weiss  US  Intensivist as a relative of a patient – retrospective piece | *My Wife’s turn in ICU* | Doctor as a patient giving an account of care  The little things like the noise, the bureaucracy, and the inattention, all contribute to the burden of suffering  and patient and family dissatisfaction  Why did it take the patient’s child to assess that having these machines in the room were not in her mother’s best interest? Perhaps, suggesting what might make the patient more comfortable during their last hours should be a role of the treating team. | **Tension:**   - Life vs Death: acceptance of death and movement towards withdrawal of life support - Attachment vs Detachment contrasting the large absence of the medical team/incorrect information with the attention and support received from friends and family; lamenting that the medical team didn’t show more interest in the patient’s comfort and that his daughter needed to question the need for ongoing support/transition to comfort care - Individual vs The System: describes personal experience as physician giving him the ability to advocate better for care that should be available to everyone – this care isn’t always readily available due to the hierarchy of the consult system |
| 2019 | Ely  US  Intensivist writing about organ donation euthanasia | *Death by organ donation: euthanizing patients for their organs gains frightening traction* | You also can’t truly conceive of the many subtle forces (to die) – invariably well meaning, kind hearted, even gentle, yet as persuasive as a tsunami – that emerge when your physical autonomy is hopelessly compromised  Instead of retrieving organs after death, organ removal would be done while organs are still receiving blood. There would be no ischemia time and organ removal would be the direct and proximate cause of death | **Tension:**   - Life vs Death: Trading one life for another, causing death to give life - Beneficence vs Non-Maleficence: Is it okay to “do good” to an organ recipient at the cost of killing another while organs are still in a human being? Does that change if the second human being *wants* to die? - Individual vs The System: contrasting perceived autonomy and lack of coercion with the real feeling of societal coercion for people inherently devalued by the system, especially those with perceived mental autonomy but lack of physical autonomy |
| 2019 | Brown  US  Commentary by senior intensivist | *The number needed to mourn* | But treatments are intense, disfiguring, and emotionally draining. I worry that the burden of therapy on clinicians looms quietly larger in decision-making than it should.  An NNT of 5 means that we must try and fail to save four patients for every one we save. We will actually hope a patient will survive, fight for recovery, and then witness their death. Four times. This painful reality may distort our vision. There are better ways to manage our grief than therapeutic nihilism.  With humility, patience, and attention to our own mental health, we who treat people at high risk for death must stretch to understand better the worlds they inhabit as they confront serious illness. | **Tension:**   - Life vs Death: describes the goal of intensive care treatment being to save life as much as is appropriately possible, that many intensive care stays are battles to stave off death - Science/Technology vs Humanity**:** contrast of epidemiological NNT with the author’s NNM – a comparison of statistics with the emotional burden of treating patients to the best of our human ability |
| 2019 | Kompanje  The Netherlands  Intensivist early in career, retrospective piece | *Parting is Such Sweet Sorrow* | A young clinician reflects on an encounter with someone losing their love and lessons for his life  “Parting is such sweet sorrow”. He asked me how I interpreted the meaning of that phrase. I explained: “There will come a day that you will part from your loved one. That will be a sorrow. But when it is with good memories, it will be a sweet sorrow”.  Every night can be our last. Make sure that the final goodbye will be a sweet sorrow. I learned this valuable lesson that night, 25 years ago. | **Tension:**   - Life vs Death: existing close to death with near-total cessation of brain function but continuing with persistence of enough function to preclude brain death - Attachment vs Detachment: being touched by the words of a provider and seeing the ”empathic filter” break down upon hearing those words repeated/immortalized |
| 2019 | Garcia-Salido  Spain  intensivist and trainees – retrospective reflection in prose format | *A thirty second pause* | A way to participate in relatives grief and honor/ respect the life and death of a patient  “We stayed there to show respect. To recognize the girl who was there. To remember her life and laughs. Through silence, we tried to emphasize the brevity and importance of that moment. Because she deserved it as a human being.” | **Tension:**   - Life vs Death: struggling to bring patients back from the jaws of death, prolonging life with resuscitation - Technology vs Humanity: contrast of the many medical treatments/therapies initiated to prolong life and reverse the disease process with the removal of that technology and the “30s pause” at end of life to recognize, cherish, and commemorate humanity |
| 2019 | Vitale  Italy  Four intensivists, fiction/satire piece | *An intensive midsummer night’s dream* | Sarcastic description of dying body and futility of care provided  Tragicomic ritual of a family for a dying man, that author at the end accept that despite the unusual scene is out of love.  Working in ICU: A dream or a reality?  Whoever you are, wherever you come from, love is  what guides people’s actions in an ICU, and every love  has its language, every love has its scene, every love its  poetry.  Love is freedom! Love is respect! Love is life!  I am not sure that what I “lived”  tonight was real or not, as it is for our patients in  ICU, suspended between life and death;  What is an ICU?  Trust in man?  Hope in God?  Patience for Godot? | **Tension:**   - Life vs Death: existing in the balance between life and death, the “Dream World” between to planes - Ordinary vs Extraordinary: comparing the surreal, dream like world of the ICU to the mundane normal life, how sometimes it can be difficult to tell dream from reality for the patients and for the providers |
| 2019 | Carenzo  Italy  Intensivist working with MSF – Narrative about an evening at work | *This is not a drill* | Another two children arrive, young, perhaps 3 years old, wrapped in a blanket; ashen, they too are covered in dust, and surely already dead. May they rest in peace. There is no time to take this in, there are still things to do for the living.  I am surprised by the fact that the mass casualty did not surprise me nor did it rattle me, and that I unconsciously worked as if nothing out of the ordinary was happening around me. | **Tension:**   - Attachment vs Detachment: describes maintaining enough detachment to preserve rational thought amidst the chaos of mass casualty - Life vs Death: quick triage decisions, fighting to save as much life and do as much good as possible - Extraordinary vs Ordinary: surreal, trauma environment in a war-torn country with little technological and physical support contrasted with the resource-rich ICU environment we’re used to |
| 2019 | Hernandez  Chile  intensivist – retrospective reflection on patient | *The man from room number seven* | We stopped rounds in front of room number seven as a signal of respect.  We felt so sad and empty. So many weeks with us, and we barely knew this man—his dreams, hopes, and fears. Almost nothing for a life gone forever. Maybe just a touch of Coltrane that kept floating around | **Tension:**   - Attachment vs Detachment: describes the irony of being so alone with so little connection despite having been surrounded by people during the weeks admitted to ICU; connection/immortalization through art – using an old poet that died young as a representation of this young, dying artist |
| 2019 | Yan  Canada  Trainee memoir and commentary | *Her ICU Bed* | Authors struggle with consequences of the decision we make in medicine, life saved but what life is that:  A fixed-eyed girl of twenty and one,  “It’s not her time,” everyone said.  A full month since the hit-and-run,  We gave her a life, in an ICU bed. | **Tension**:   - Life vs Death: prolonging life against death at the cost of quality of life, perhaps death would be kinder, more appropriate – existing in a state of limbo, purgatory of non-perception with no interaction - Technology vs Humanity: keeping the patient alive with machines at the cost of having lost the personality and interaction that made her the human she was |
| 2018 | Cortegiani  Italy  Trainee, Intensive care  Reflecting on conversation with relative | *Gulliver’s travels in the intensive care*  *unit* | Through analogy of “Gulliver’s …” a slow, death or a life in ICU where the person is transformed is worse than death  Only in this island of Luggnagg was the appetite for living not so eagerl from the example of the Struldbrugs before their eyes… The Struldbrugs… were not able to hold any conversation with their neighbors the mortals… They were the most mortifying sight I ever beheld.  … I kept my promise. It was about the Struldbugs story, right? The men and women who have denied dying? – Yes doctor. And, concerning my dad, this room looks like Luggnagg | **Tension:**   - Life vs Death: prolonging life despite death perhaps being kinder, death as a release from a state of “unlife” - Hope vs Despair**:** Re-framing expectations to preserve hope even in the context of seemingly imminent death - Technology vs Humanity: contrasting objective data with human emotions; validating the feelings of the patients |
| 2018 | Petrucci  Italy  Intensivist | *Beyond bleeps and alarms: live music*  *by the bedside in the ICU* | When awake, everything is foggy, permeated only by pain when medications wear off. The reality of awakening in the ICU is still beyond anyone’s comprehension. Confusion and fear are the emotional responses.  Live music performance seems like something unexpected in the ICU. But to relatives it is a sign that the ICU team really takes care of all aspects of patients’ well-being | **Tension:**   - Technology vs Humanity: contrast of the cold, monotonous beeps and alarms of the ICU with the provision of live music to create a more humane environment that could reduce delirium |
| 2018 | Shrestha  Nepal  Intensivist reflecting on bility to save his mom’s life because he is a doctor (poor care for the rest of the population) | *A walk after hemiplegia* | The friends, relatives, and moreover the health care workers and doctors started to praise a brave son for making this miracle a reality. However, I could not stop asking myself a series of questions. Would not every don of daughter want their parent to be managed as recommended by international guidelines? Why have the guideline recommendations remained applicable only to a limited proportion of the global population residing in the western world? | **Tension:**   - Pride vs Guilt: Happiness and pride at being able to save his mother contrasted with the guilt that this outcome would not have been possible if not for his status/access to and within the health care system - Individual vs The System: ability to save his own mother contrasted against the inequity of the health care system in which he practices; why does this unfairness persist? Should not everyone have access to life-saving care? |
| 2018 | Elia  Italy  Intensivist | *The patient who fell off a skyscraper* | This is the real story of a man who fell off a “chronic disease skyscraper”, stumbling over his COPD. Every floor is one of several significant illness episodes. On his way down passing each floor, doctors got used to reassuring him saying: “So far so good”.  It is not true that how you fall does not matter. The way we land does depend on the way we fall. Our job is not confined to slowing the rate of descent. It is also our concern to make clear to patients and families what is going on during the fall and equip people for a landing as smooth as possible. | **Tension:**   - Hope vs despair: describes how poor communication can alter expectations and create false hope, making people grossly unprepared for dealing with reality |
| 2018 | Frank  Austria  Intensivist | *HES: time to change my mind?* | I will continue to follow  the scientific discussion and may change my mind, as  every clinician should do sometimes.  there is one reason to use HES  and two reasons not to do so. | **Tension:**   - Individual vs The System: comparing individual patient assessment and personalized medicine with published “one-size fits all” guidelines from RCTs |
| 2018 | Maringer  US  A wife’s experience in the ICU | *There’s more to medicine than machines* | To a lay person, ICU patients look like science experiments—immobile and hooked up to so many beeping machines.  realized that choice was an illusion.  To the family of someone who is critically ill, the ICU feels like you have entered an alternative universe. Time feels suspended because the lights are always on and the machines are always beeping  Perhaps most importantly, I learned that medicine is as much an art as a science. I kept asking questions that I felt should have answers. But there aren’t always answers.  Sometimes doctors do what they think or hope will work, but aren’t sure. | **Tension:**   - Technology vs Humanity: describes how after connection to ECMO that her husband became “an experiment”, not actually a human being underneath the machines - Extraordinary vs Ordinary: contrasting the surreal environment of the ICU with that of ordinary life and how that the ICU environment eventually became the “new ordinary”, turning life outside the ICU into a surreal experience - Life vs Death: account of how essentially all choice was stripped away – ECMO was the only way to stave off death and even that wasn’t a guarantee - Attachment vs Detachment: describes reminding health care providers that her husband was not just a case and that the compassion shown to her was the only way she was able to make it through the ordeal |
| 2018 | Bein  Germany  Senior intensivist | *Successful repair of aortic dissection,*  *but no more playing with the grandsons* | The man’s dearest wish is to spend a couple of years with his grandsons, to watch them grow up, and to reward them for good school reports. While talking about the risks and a successful repair, oscillating between fear, hope, optimism and helplessness, the surgeon takes the time to let the patient ‘participate’. But is a true participation possible?  So the question remains: are we as physicians responsible for such a ‘result’? We could easily respond with “No, we just followed our medical guidelines….” But upon deeper reflection, we might not be so sure. Perhaps we should not think about it too much; otherwise, we might feel uncomfortable.  Modern medicine allows unimagined possibilities to save one’s life and keep death at bay. However, the same medicine opens up a new minefield of grey zones and limitations: The patient may end up living a life that he did not strive for and that he did not imagine when opting for surgery. But he has to accept it. | **Tension:**   - Choice vs Chance: was it the right choice to proceed with surgery? Or would it have been better to take a chance at delaying repair in an effort to continue a good quality of life? - Life vs Death: Is it right to prolong life if it’s not the life we envisioned ourselves living?   Contrasting the regret felt towards the surgery with the pressure to be grateful for extended life |
| 2018 | Kompanje  The Netherlands  Intensivists memoir and commentary | *Burnout, boreout and compassion*  *fatigue on the ICU: it is not about work stress,*  *but about lack of existential significance*  *and professional performance* | To her horror, she had become a cynic to the suffering of her patients.  We fix the symptoms of chronic sick patients who don’t have the will to heal themselves.  The roots of burnout, boreout and compassion fatigue lie in people’s need to believe that their lives are meaningful, that the things they do are important, make sense and give existential significance. When people feel to have failed, being insignificant and making no difference, they start to feel helpless, hopeless and they crash | **Tension**:   - Attachment vs Detachment: fulfillment and purpose are described as safeguards against burnout and compassion fatigue; when people are cynical and apathetic, reminding them of their purpose, positive impact, and providing them with recognition of their accomplishments may spur a transition from burnout to self actualization |
| 2018 | Elia  Italy  Intensivist | *Subtle Trajectories* | this gives us the illusion of being able to fully control the biological aspects of our patients’ existence. It almost appears that their lives depend on our interventions and decisions.  if something goes right, this is thanks to us and to our choices, and on the other hand, if something goes wrong, this is because of us and our bad decisions … A sort of self-certification of almightiness  A first step forward is accepting the uncertainty; the next is finding a means of explaining it to the patients and their family. Most probably, we are less important than we deem ourselves to be. | **Tension:**   - Extraordinary vs Ordinary: describes our belief in almost superhuman abilities where our choices mean the difference between life and death, then calls attention to the contrary reality where our choices likely mean less than we think they do |
| 2018 | Nguyen  France  Three intensivists reflecting on patient’s experience | *Suspended in time and space* | His thoughts are trapped in a closed circuit, events lack meaning, his hands are not active, and he himself, as a Person, finds it hard to exist.  We are like in a submarine? Mr. P nods. He shows that there are no windows, no natural light, and no knowledge of night or day—no landmarks.  He feels lost not only in time but also in space.  has lost his temporal and spatial landmarks. He feels disconnected from himself and the world.  Active listening and effective communication should thus be encouraged, not only to benefit ICU patients, but also to benefit clinicians themselves who may suffer from stress and burden. | **Tension:**   - Attachment vs Detachment: describing the lack of ”ordinary” landmarks (i.e. tasks, time, thoughts, and other day-to-day stimuli) creates an isolating feeling that was partially overcome by human connection and active listening - Extraordinary vs Ordinary: contrasting the distortion of reality inside the ICU (i.e. altered perception of time and space) with ordinary life where time passes more quickly and orienting oneself in space is easy |
| 2017 | Quintel  Germany  Intensivist reflection on his journey and lessons | *“I feel how you feel”: reflections*  *about empathy in the relationship between ICU*  *physicians and relatives* | trying to explain the actual status of the patient and you realized that your counterpart tried to follow your words but remained immersed in a sea of fear, pain, doubt, uncertainty— a world of conflicting emotions. You stopped because all of a sudden you realized that some of these emotions had become your own—at least in part you felt what she, he, they felt.  We simply found the balance between empathy and factual needs.  they view the ICU environment as something alien, governed apparently by logic and technical rules where emotions seem to be excluded because they might even endanger “effective” and lifesaving therapy.  In my view the truly good physician lives in balance between thoughtful unemotional actions and honest empathy; both require readiness for life-long learning. | **Tension:**   - Technology vs Humanity: Balancing objective assessment/technical data with human emotion and compassion; contrast of cold, technical, inhuman ICU with the warm, feeling humans that populate it |
| 2017 | Koch  US  Intensivists’ commentary on assessment of ”a good life” | *What does it mean for a critically ill*  *patient to fare well?* | Determining what makes a good life for individuals with distinct functioning and capabilities on account of illness or injury is a powerful and neglected challenge in intensive care medicine.  Clinicians, in conjunction with surrogates, are frequently faced with judgements regarding the burdens and benefits of continuing artificial support—and life itself. | **Tension:**   - Extraordinary vs Ordinary: making decisions/assessments that seem beyond human capability with only human abilities |
| 2017 | Naretto  Italy  Intensivists sharing their experiences | *Storeroom 99: a place for words*  *to support families of ICU patients* | Without numbers, without statistics and evidence based stuff, medicine does not seem right; it’s incomplete, awkward. How can we get resources if we can not prove that it works? Well, we know talking is a good thing, do we not? Words can heal. Relationships are crucial for a healthy and happy life. Sometimes this kind of support is much better than a hundred medications; careful listening is more effective than a disability score. And we can give this support; because we were there, in the storm,  We do not have proof it works. No numbers, sorry! But do we need the chemical formula of a strawberry to taste its sweetness? | **Tension:**   - Attachment vs Detachment: contrasting the narrative with the objective, evidence-based assessment; human connection as a way of healing in addition to the available technology/medication |
| 2017 | Bein  Germany  Intensivists memoir and commentary | *Empathy: some thoughtful reflections*  *among a favorable attitude* | Doctors always need to reach a balance between connection and distance in their relationships to patients. The basis of empathy is the fundamental acceptance of the existence of the ‘Other’.  “I personally could be a fan of a well-defined (and educated) empathy, combined with competence and respect as ingrained in a self-confident (but not arrogant!) physician or nurse.” | **Tension**:   - Role as Physician vs Role as Patient: contrasting responsibilities as physician with the experience of being cared for as a patient - Attachment vs Detachment: contrasting empathy with objectivity; are they mutually exclusive? If compassion comes at the expense of competence is it really that important? Appropriate compassion should be balanced with high competency and mild detachment to preserve objectivity |
| 2017 | Raffa  Italy  Intensivists commentary on health issues of migrants | *Migrants crossing the Mediterranean*  *Sea: an opportunity or a duty?* | We are sick at heart to hear international news reporting  people dying in the Mediterranean Sea and to see  pictures of children lying dead or dying on the shores of  Europe, images that have become a symbol of human and  social failure  Health care providers have a duty to provide the best  possible care with a reasonable use of resources, assigning  patients to the adequate level of care in light of their  clinical condition, and not simply because of their status  as “outsiders”. | **Tension:**   - Extraordinary vs Ordinary: responsibility to transcend ordinary responsibilities associated with clinical work and advocate for care to disadvantaged populations - Technology vs Humanity**:** providing advanced technological support because it’s the human thing to support vulnerable populations |
| 2017 | Bose  USA  Intensivist non fictional essay on death of a patient | *Healing ourselves* | This meant the loss of a 36-year-old under my watch.  Being human first, our cognition and actions are invariably influenced by our emotions.  Doubts and second guesses filled their voices. I did not know how to answer.  These emotional rollercoasters are played out with the greatest vividness and frequency in the intensive care units. The extreme acuity and abrupt changes in clinical situation make intensive care units the most fertile grounds for such upheavals. We come to realize that we shall win most battles, but also lose some. Losses hit close to our hearts and we perseverate. | **Tension:**   - Life vs Death: inevitability of death despite efforts to maintain life. Illusion of control over the outcome, taking personal responsibility for an otherwise inevitable death - Extraordinary vs Ordinary: superhuman demands that often need to be met by only human abilities - Emotion vs Objectivity: desire to remain objective and “perfect” contrasted with emotional decision making - Technology vs Humanity: contrasting objectivity/physiology that is required in decision making with human connections forged between caregivers and their colleagues/patients |
| 2017 | Kompanje  The Netherlands  Intensivist commentary on teaching healthcare providers | *Will we become more compassionate or empathic intensive care professionals by watching films and documentaries about suffering?* | Do we, as professionals working in ICU, experience more mental pain when we are watching a film or documentary or reading a fictional book about suffering than when we experience this in real life? No, it seems that we experience empathy in another way.  anguish, in compassion we only show concern. Why do we want to read a novel or watch a movie about the pain and suffering of others in the first place? That we appreciate experiencing the kinds of emotions that we tend to consider unpleasant in daily life, like death, end-of- life, suffering, may seem rather illogical. This is called the ‘paradox of tragedy’. | **Tension:**   - Art vs Science: contrasting the learning that can be had from art against that which is experienced in the clinical setting; the use of art to highlight and move on from unpleasant emotions |
| 2017 | Garcia-Salido  Spain  Intensivists reflections on a comment made by a patient | *Knock, knock, knockin’ ….on Critical Care’s door* | I do not understand why we could not stay with my father as now I stay with my son here. | **Tension:**   - Technology vs Humanity: contrasting the research heavy world of adult critical care with the family-centred approach at the PICU bedside |
| 2017 | Isaac  USA  Intensivists sharing their experience through a commentary | *How to respond to an ICU patient asking*  *if she/he is going to die* | A question like this can be deeply uncomfortable for clinicians. It obliges us to deliver potentially devastating news about prognosis directly to an ill patient. In the process, we must face our own apprehensions about navigating difficult conversations and perhaps even contemplating our own mortality  In addition to delivering serious news, clinicians often struggle with maintaining the balance between hope and truth-telling in the face of uncertainty.  As clinicians treating patients at the end of life, we seek to understand those for whom we care and in that understanding,  we find our opportunity to heal when we cannot cure | **Tension:**   - Life vs Death: preparing people for death despite the fear that one may have of it - Hope vs Despair**:** Re-framing expectations to preserve hope even in the context of seemingly imminent death - Technology vs Humanity: contrasting objective data with human emotions; validating the feelings of the patients |
| 2017 | Alonso-Ovies,  Spain  Intensivist | *Children under… Not Admitted* | JMs eyes are focused in his small treasure. The force of that mischievous looks seems to be the only thread that keeps him attached to life, as the hope to see her again | **Tension:**   - Life vs Death: inevitability of death coupled with living on through loved ones - Individual vs The System: inability to fulfill a dying man’s wish due to the restrictions of the system |
| 2016 | Lazaridis  USA  Discourse on current organ donation policies and the institution of radiacal organ-conscription policies to improve donation rates | *Transforming ICU Death into Life – Radically More* | Careless clinical thought in ICU certainly costs lives; we should equally realize that careless moral thought is as unacceptable, and that refusing to even discus “radical” proposals in order to increase availability of organs can be careless, immoral, and lethal.  Patients who meet neurological criteria for death should have their organs made automatically available… Organs here are considered as a type of public good… and vetoes to their use either from deceases or their family are dismissed on the basis of the following assertions:   1. Organs are of no use to the deceased 2. Organs from the deceased are of no use to the family 3. These organs will inevitably destruct if not harvested   The life-preserving claims of the living autonomous persons trump ante-mortem autonomy claims | **Tension:**   - Life vs Death: ability to turn death into life via organ conscription from NDD patients; autonomy of the living trumps that of the dead - Beneficence vs. Non-Maleficence: utilitarian, deontological, consequentialist approach to maximizing benefit, and societal justice against the autonomy of the dead to keep their organs |
| 2016 | Gobert  France  Neurologist, Respirologist, Intensivist, reflection on a complex case | *Buying time to save a life* | Up to what point could the confidence we have in such prognosis—in clear conscience— legitimately modify the future of a piece of the mankind we have in our charge, represented by a single individual destiny?  "But what is the cost we could accept?" may ask both society and institutions. in waiting or stopping patient management are we really reading the future in our medical devices? Or are we just writing ourselves the end of the story, in choosing one of these two opposite actions? | **Tension:**   - Life vs Death: Limited power of medicine to save/prolong life - Individual vs The System: tension of the benefit to patient society - Superhuman vs human: Imperfect prognostication to guide high stake decisions, ability to irreversibly change lives based on our choices |
| 2016 | Ely  USA  Intensivist non fictional essay on patient’s end of life | *Swimming pool in the ICU* | Amidst all the surrounding insanity of the hospital that day, diving deeply into Bennie’s life through his baptism on the breathing machine allowed all the rest of us to be reborn too. Being “with” him in that pool, and rising with him out of it, we walk into other’s lives better prepared to serve. | **Tension:**   - Life vs Death: shift of focus from prolonging life to accepting death and improving quality of remaining time alive - Technology vs Humanity: impracticality of baptizing a ventilated patient but success in doing so through communication, perseverance, and respect for the patient’s belief system |
| 2016 | Sarnaik  USA  Oncologist, mother, and patient reflections over events many years ago | *Anne Marie’s Christmas* | The intensivist, the ICU fellow, the nurse, and the respiratory therapist blended with the walls, unnoticed but content, experiencing this improbable scene.  Risks were taken. But a promise was kept, a dream came true, and a little girl was able to celebrate Christmas with her family.  The only thing that was keeping Anne Marie from celebrating Christmas at home with her family was that wretched endotracheal tube | **Tension:**   - Technology vs Humanity: medical technology as a barrier to experiencing human contact with the family during the holidays - Beneficence vs Non-Maleficence: the good at getting a child home to celebrate with her family vs the potential harm that could occur from additional risks outside of the ICU setting - Ordinary vs Extraordinary**:** the importance of making room for ”ordinary” experiences in extraordinary circumstances, and how those experiences may become extraordinary over time |
| 2016 | Alonso-Ovies  Spain  Intensivists non fictional essay on a patient | *ICU: a branch of hell* | Intensive care units are nowadays probably the paradigm of the ‘‘dark side’’ of modern medicine, where mechanization has displaced the patient; this person is not seen as a human being who feels and suffers, but as a complex problem to solve.  Fortunately, scientific knowledge, technology, and skills will be further developed in the coming years. Let’s try to develop our ‘‘human’’ side at the same time.  ‘‘The problem is not the pain, the pain and the  fear are. Sometimes the fear is worse that the pain, and the  combination of fear with pain… There was a time when I  thought I was dying, and, above all, there came a time  when I already wanted to die…’’. | **Tension:**   - Technology vs Humanity: objectification/dehumanization of patients as “complex problems to solve”; need to develop human side of medicine and improve compassion delivered as a part of exemplary, life-saving care; despite the life-saving technologies, there are times where the patients actively want to die due to unpacified fear/pain |
| 2016 | Vermeir  Australia  Patient’s relative an intensivists reflection on the care | *Not out of the Woods* | While it is obvious that all the attention is focused on the patient, it is important to acknowledge and recognize that the loved ones are those who are often left to suffer in silence. We all undertake and express this silent journey  in different ways. | **Tension:**   - Attachment vs Detachment: taking time to engage and communicate with family vs remaining detached to deliver completely objective care to the patient |
| 2016 | Van Keer  Belgium  Researcher’s poem from a patient’s perspective | *Intensive suffering* | No doctors who speak Turkish, nobody I can understand; Except that one nurse; Who daily holds my hand  Seeking distraction in writing; But only for a while; Why did life become so very frightening? | **Tension:**   - Attachment vs Detachment: detached from providers who couldn’t communicate with special attachment to the one who could offer human contact through a shared language.   Maintaining cheerful demeanor in front of family despite internal feelings of desperation and terror. |
| 2016 | Heras La Calle  Spain  Intensivist, non fiction essay reflecting on experience with end of life | *An emotional awakening* | ‘‘Neither as a man nor as a doctor can you get used to seeing our fellow man die.’’ I have also witnessed death way too many times, much more than the rest of the population.  We tend to feel that we have failed in the application of our knowledge, or because of unrealistic expectations of what is humanly possible.  …countries where death is seen as something normal, a natural process that is simply accepted as such.  … plan our birthdays or weddings months in advance, yet we ignore death or how we want to die or be treated in our last hours on this earth!  help our patients and their families to understand that death is part of the life process, to accept death for what it is without labelling it good or bad, and that it happens to all of us no matter who or what we are. That cold fact does not make it any easier, but through emotional support and genuine caring the shock and numbness can be lessened. | **Tension:**   - Life vs Death: prolonging life at the expense of a “bad death”, contrasting the “vibrancy” of life with reticence to accept the inevitability of death - Technology vs Humanity: contrasting the science by which life is sustained with the human connection wrought by good communication |
| 2015 | Polastri  Italy  Intensivist; Non fiction essay; Reflecting on experience with a patient | *Can we walk?* | Days later, the girl sees me through the cubicle’s glass, passing into the ICU. She jumps up from the bed, ready to stand up and go out. As I walk toward the sliding door, I look at her smiling and calling to me from beyond the glass, ‘‘I’m ready, can we walk?’’  She sees her image in the mirror for the first time after several days and laughs… Her expression communicates happiness, fear, uncertainty, and determination.  I help her put on her socks and soft shoes, although she does not like the fold of the socks and asks me to fix them. As I am doing this, she is looking at me with approval and a knowing glance that she is ready to begin. | **Tension:**   - Attachment vs Detachment: highlights inherent humanity in connecting despite the barriers of glass and critical illness - Hope vs Despair: drive to recover despite uncertain, complex prognosis |
| 2015 | Vavuris  USA  Nurse Intensivist | *Am I dead?* | She asks ‘‘Am I dead?’’, a reflection of the delirium she was experiencing. It was a very poignant and emotional moment for her mother and me.  Despite her confusion, she carefully punctuated her query with a question mark. | **Tension:**   - Ordinary vs Extraordinary: contrasting the patient’s delirious experience with the reality of the moment |
| 2015 | Struwing  South Africa  Physician and relative Poem | *By your side* | It was so much easier then to hold your quiet hand  than it is now to keep your spirit standing tall.  But I am still here despite it all, always  sitting by your side, waiting to hear  your laughter sprout again. | **Tension:**   - Hope vs Despair: grim prognosis/new reality vs hope for eventual recovery |
| 2015 | Kompanje  The Netherlands  Intensivists and ethicist reflections of connection to patients | *‘I just have admitted an interesting sepsis’.*  *Do we dehumanize our patients?* | We do not want the health care providers to be suffering with the patients, but we want them to know that they suffer. Beside this, we should respond to the patients and their relatives with empathy, compassion and sensitivity. We must do more than simply ‘know’ they are suffering, but we must also alleviate that suffering to the best of our ability and, when we cannot alleviate their suffering, we should be able to be there as a witness and provide support through our presence.  So, yes, we dehumanize patients. But this is inevitable, adaptive and even morally and psychologically accept- able. As long as health care providers know that their patients are suffering and in pain, they do not have to feel it. | **Tension:**   - Attachment vs Detachment: walking the tight line between enough detachment to preserve the mental health of the provider and enough connection to the patient to preserve their humanity in spite of their illness |
| 2015 | Bein  Germany, Canada  Senior Intensivist reflecting on deah and dying on ECMO | *Extracorporeal life support, ethics,*  *and questions at the bedside: how does the end*  *of the pathway look?* | The question may come up ‘‘why is it that it is so hard to die now?’’ Is it that we have to exhaust all technologies before this is OK? The cost may be enormous suffering and a very traumatic death for the patient and the family.  The identification of the margins of human viability has left a precisely defined terrain, and modern medicine anthropology is about to leave the Kantian view of an autonomous self-reflecting individual in favour of a primary maxime: survival— whatever the cost. We feel the latter is not correct.  Is ECMO a bridge to recovery, a guarantee of a status quo, or just prohibiting dying? These situations will never be ‘pressed’ in guidelines or algo- rithms; however, our view needs to be directed in these reflections, and we need to develop strategies that address these questions. | **Tension:**   - Life vs Death: preserving life even when death is inevitable - Beneficence vs Non-Maleficence: preserving life at the cost of harming the patient/causing “violence to the body” - Confidence vs Uncertainty: remaining a confident professional despite uncertain prognoses/uncertain emotions |
| 2015 | Neville  USA  Intensivist, non fiction essay reflecting on experience with a patient | *Only human* | But if a mother wanted to be with her son, no matter what condition he was in, was it wrong of me to let that be possible? But who was my patient? I pause here and acknowledge it was the bleeding, dying boy that I continued to maintain on multiple life-sustaining modalities. I shouldn’t have let dialysis be an option.  I still wonder if his mother is haunted by her last memory of him dying in the ICU. And, today, I am writing my thoughts on paper to perhaps get some sort of relief. More than I am a critical care physician, I have realized, I am only human | **Tension:**   - Life vs. death:   Caring for the patient and preserving life despite causing harm to the body   - Attachment vs detachment: Need to make the “right” decision despite human emotions/opinions getting in the way of objectivity - Parents Wishes vs What’s right for the patient: continuing futile measures for sake of the parents vs the patient |
| 2015 | Benbenishty  Israel and USA  Nurses and researcher in Intensive care commentary | *Non-verbal communication to restore*  *patient–provider trust* | ‘‘the use of touch is the universal language of caring.’’ Touch in health care has two modes: (1) touch during interventions, (2) conveying emotional messages. Since touch is required of most nurses during patient care, a nurse must retain awareness of what message her touch conveys. | **Tension:**   - Attachment vs Detachment: touch as a way of overcoming the barriers between patient/provider and communicating trust |
| 2015 | O’ Callaghan  Australia  Reflections of a patient journey in ICU | *From the intensive care bed...we need to hear*  *and listen* | The daily ritual of the consultant swinging by with his  entourage was an experience. I was saturated with drugs  but could still hear and see.  I was intubated and could not communicate, but I could see and hear. I was often left to generate my own interpretation which was clearly fraught with danger.  In my ICU cubicle was a suspended clock staring straight  back at me. If you want to know how long a minute is, let  me confirm it’s a lot longer than 60 seconds. When all  you can do is lay in bed staring at a clock, every second  feels like an hour and every minute feels like a day. | **Tension:**   - Extraordinary vs. Ordinary: perception of time moving more slowly due to preoccupation with the clock, altered perception of reality due to surreal ICU environment - Attachment vs Detachment: detachment from care providers, treated as a disease process rather than a fellow human being |
| 2015 | Shaw  Switzerland  Intensivist and ethicist opinion on organ donation | *Organ donation is the right decision: a delicate*  *truth* | It is a delicate truth, but organ donation is the right decision. How else could you describe a decision that saves lives and prevents suffering at virtually zero cost? People might not like it when they’re told that refusal to donate is the wrong decision, but there’s no other way to characterize it. Everyone has a right to make their own decisions about donation, but no-one has a right not to be told that it’s a bad one, all things considered. | **Tension:**   - Autonomy vs Beneficence: right of the individual to choose what happens to their organs vs ensuring procurement for the “greater good” of the patient population - Autonomy vs Non-Maleficence: right of the individual to deny organ donation vs the harm that is caused to patients by withholding viable organs that could save lives |
| 2015 | Kompanje  The Netherlands  Intensivist and ethicist opinion on organ donation | *Sounding board: is mandatory recovery*  *of organs for transplantation acceptable?* | For relatives of possible organ donors, the balance between a peaceful and dignified death and the social obligation to help another sick patient is delicate. Mandatory retrieval of organs has no place in this line of thought. Never. | **Tension:**   - Autonomy vs Beneficence: right of the individual to choose what happens to their organs vs ensuring procurement for the “greater good” of the patient population - Autonomy vs Non-Maleficence: right of the individual to deny organ donation vs the harm that is caused to patients by withholding viable organs that could save lives |
| 2015 | Villa  Italy  Intensivists commentary on physiology | *Waves…from the inside* | Each viewpoint may influence others, leading to situations in which the scientific speculation on pathophysiology and the application of specific techniques evolve toward identification of the humanistic individuality of the patient. | **Tension:**   - Technology vs Humanity: using science, technology, and understanding of pathophysiology as a way of connecting to the patient’s individual experience/manifestation of illness |
| 2015 | Lazaridis  USA  Intensivist reflection on an encounter with a patient | *Where was I when I was in a coma?* | where she is now, and if she is back in the same place? It misleadingly sounds like a question about location, yet this is a question about being. With that in mind the original question is reframed as ‘‘what (or who?) was I when I was in a coma?’’  I explained then that the more careful answer is that during coma she was no one; she gave me a puzzled look, and I quickly added that her person was ‘‘carried through’’ the thoughts of her loved ones and of the medical team that cared for her.  Before leaving her I said I was really glad she was doing so much better; she smiled and answered remarkably: ‘‘and I am glad to be someone again’’. | **Tension:**   - Technology vs Humanity:   During coma, without thought/ability to express one’s individual personality they are in fact “no one”. Meanwhile others substituted them. Only upon regaining consciousness were they “someone again”. |
| 2014 | Benbenishty  Israel  Senior nurse clinicin; Non fiction essay | *A meaningful closure* | That ‘‘some day’’ is today.’’ R nodded in silence. Prof. Anner continued: ‘‘I am here with you. I won’t leave you.’’  ”I was able to identify my role and guide this family into their final, most crucial closure – you only get one chance to get it right. […] my sense of accomplishment in traveling the closing pathway of Mr. R’s life was gratifying.” | **Tension:**   - Life vs Death in end of life process – facilitating dignified death while witnessing the humanity of a patient’s final moments - Control vs powerlessness/Choice vs chance – inability to treat/”Save” the patient, but able to control response in final moments |
| 2014 | Denegri  UK  Leader in involvement of patients in research  Poem from patient’s perspective | *The research poem* | So here I am  Neither subject nor guinea pig  With no letters after my name.  For when dusk does night betray  I want to be able to say  I made a difference for me, for you, for us. | **Tension:**   - Technology vs Humanism – the patient as a person and not just a “guinea pig” to be studied - Permanence vs impermanence – the will to leave behind a permanent legacy, to avoid being forgotten |
| 2014 | Fenn  UK  Patient essay on their ICU admission | *Coma alarm dreams on pediatric intensive*  *Care* | My head injury is a series of disjointed images in my  mind.  My experience of the time under sedation can be split  into two. There was what I could perceive of the real  world around me, and then there was my dream world. | **Tension:**   - Extraordinary vs.ordinary   awareness of bedside communication/alarms that escalated to fearful, surreal “dream state” Preserved, vivid memories of surreal experiences with subsequent PTSD from ICU admission |
| 2014 | Kumar  India  Poem, written by intensivist describing the dying | *Plight* | Hoping against hope in the sea of despair where the hope  only floats.  Breathing yet breathless, living yet lifeless.  Caressed by the icy hands, freed from the burning breaths,  peacefully laid  No pains—No agony,  No sufferings—He’s dead | **Tension:**   - Life vs Death: maintenance of life on support despite the inevitability of death - Suffering vs Relief: harm/suffering of being on life support and the “relief” of death - Hope vs Despair: “locked hopes” despite the futility of the situation |
| 2014 | Shaw  Switzerland  Ethicist and Intensivist recommending a way to direct their loved ones about his wishes. | *Creating my personal organ donation directive* | if I do happen to die in a way that makes donation a possibility, the last thing I want is for my family to veto donation, which happens quite frequently. I am on the organ donor register because I want my organs to be used to save other people’s lives and to improve people’s quality of life. If my family vetoes donation, three or four people whose lives could have been saved will die, | **Tension:**   - Autonomy vs beneficence/non-maleficence: doing the right thing to achieve the greater good; - Control vs Powerlessness: ensuring that the author’s wishes are followed even after his death (when he has no power to argue his decisions |
| 2014 | Rier  Israel  Medical Anthropologist reflecting on his experience with healthcare system | *From three sides now: reflection of an ICU journey* | my hospitalization represented the start of a journey,  weaving together the personal and professional.  Make some sense of what the doctors were—and were not saying, and to fill in the many blanks their explanations  often left.  Overall, I played a form of ICU ‘‘Kremlinology’’, trying to piece together doctors’ sometimes-cryptic, fragmentary explanations, body language, and various other clues into some semblance of a picture of diagnosis and prognosis.  there is a strict protocol of gowning, gloving, and scrubbing on entering and exiting the room. Such policies have obvious benefits for infection control. Who can argue with that? But, thinking back to my own patient experience, I noticed something that has not attracted much discussion: such arrangements can deny patients crucial company and emotional support. | Role reversal: from studying ICU to becoming a patient or a relative  **Tension:**   - Hope vs Despair: maintaining hope for the patient by putting an “optimistic spin” on his situation despite the severity of his illness - Extraordinary vs Ordinary: extraordinary environment made to feel as normal as possible through human interaction - Personal vs Professional: role reversal – from studying ICU to becoming a patient in an ICU, parent/grandparent in NICU |
| 2014 | Butka  USA  Poem of a nurse in Intensive Care Unit | *Night shift: Intensive care* | the sick, whom we shepherd  through the shards of night,  dream their jittery dreams, unconscious,  scissor-step from light to nightmare  and back again.  Not everyone lands on a warm and happy planet the automatic doors clanging open, greeted by family in a pink haze | **Tension:**   - Hope vs Despair: hope for full recovery vs the reality of a poor prognosis - Extraordinary vs Ordinary: surreal ICU environment met by the mundane environment of every day life |
| 2014 | Castro  Chile  Intensivist non-fictional essay on his experience with a patient | *I wished she had meningoecephalitis* | Everything could be better than expected. There was some hope, but who is in need of hope? The patient? The family? The doctor?  As physicians, many times we want to turn around the inescapable course of the events. Somehow, someway, we get some hints that seem to endorse our innermost longings. Otherwise unintended happenings or smart and right colleagues’ opinions that, when thirsty for hope, we regard as providential.  Reality always stood in front of my eyes, but honestly I did and still wish that she had meningoencephalitis. | **Tension:**   - Hope vs Despair: desire for diagnosis to be favourable/recoverable vs the reality of a psychiatric diagnosis with poor response to treatment – responsibility to patient to get true diagnosis vs desire to give the patient a favourable prognosis with good response to treatment - Detachment vs Attachment: need for objectivity in the diagnostic approach vs personal feelings around the tragedy of the diagnosis |
| 2014 | Friedman  Brazil  Senior Intensivist reflecting on end of life | *Time to die!* | Who can determine the best time to die?  A week after, her husband sat beside his wife, took her hand lingeringly, and probably whispered lovely words. After long minutes, he removed the wedding ring from his wife’s finger, walked in our direction, thanked us, and asked that all life support could be withdrawn. | **Tension:**   - Life vs Death: Delaying death in order to commemorate a life lived together with her husband   Ability to choose time of death even though mortality was ultimately inevitable   - Care vs Suffering: care for the family/relationship between husband/wife vs prolonging the suffering of someone on life support - Technology vs Humanity: continuing “futile” life support in order to reach a human conclusion |
| 2014 | Maclaren  Singapore & Australia  Intensivist reflections | *Music and Medicine* | Every art form is solely about communication, including the art of medicine. This communication, being able to reach out and meaningfully touch someone else’s life, is a very special privilege.  When we meet patients or parents of critically ill young children at major crossroads in their lives, crossroads inevitably overcast with sorrow and uncertainty, we have an opportunity to make a unique connection with them, one not found through any other means in life.  emphasis on single rooms and partitions, and the intensity of the workload can shift the focus of intensive care specialists away from trying to reach out and make meaningful contact with their patients, and risks oversimplifying everything to numbers and applied science…technology can distance us from the rest of humanity  Although compassion is vital for patient care, it increases the emotional stress on the clinicians and takes a toll smothering these emotional responses is hazardous.  playing and listening to music still continues to have a pivotal role in my life, constructively channeling emotional stress and helping put life and death in perspective. | **Tension:**   - Technology vs Humanity: contrasting the technical, objective nature of medicine/medical assessment with the human connection between patient/provider - Attachment vs Detachment: contrast between the connection/sense of fulfillment created by art and the distance created by technology   Expectation of medical practitioners to be objective, empathetic, and perfect vs the draining nature of constantly being open to connection/compassion fatigue |
| 2014 | McAdam  USA  Nurse and doctor clinician’s in ICU reflecting on their experience clinically and research on end of life | *Regret* | We all know what it means, we all experience it, and we  all know how it feels. But what is it about death and dying  that seems to bring feelings of regret in people?  We frequently deal with dying and death, sometimes on a daily basis. But just because we have that experience and know what to expect does not mean we do not have our own regrets that find their way into our personal lives as well.  Maybe if we understand and learn from that, we can help families forgive themselves, and we may become more forgiving of ourselves. | **Tension:**   - Forgiveness/Contentment vs Regret: Burden of emotions related to being close to death – the need to leave behind a “good” legacy - Life vs Death: sudden, permanent nature of death and the lack of control over its occurrence vs the control of our reaction to it |
| 2014 | Sanchez  Chile  Intensivist reflecting on his experience with a patient | *The final voyage* | He has been gone now for a couple of years. Sometimes  I remember him, especially on misty autumn days. I  do not know why but his name evokes a specific landscape  far away  I have been an intensivist for so many years now but never stop thinking that in the window of every patient’s experience you can find the signals that can lead you to become a better human being but also an intensivist who cares, who looks, and acts differently. It is crucial to find the balance between compulsive technological care or just listening to the patient’s dreams.  However, no more invasive technologies are acceptable. Dignity is my only goal now.’’… There were tears but no despair.  Crucial to find the balance between compulsive technological care or just listening to the patient’s dreams. This was Don Dago for me, a man who lived and died like he wanted to. | **Tension:**   - Technology vs Humanity: balancing technological support with the patient’s human wishes/desires at end of life - Teacher vs Student: balancing the role of oneself as a teacher of others while learning from patients - Life vs Death: the legacy of a “good” life vs the permanence of death - Choice vs Chance: controlling the manner of death despite being unable to control when it happens   Role reversal: patient as a teacher about how to live and how to die |
| 2013 | Galvez  Chile  Intensivist writing a poem from a dying patient perspective | *A Human Plea* | But my humble gratefulness will be for that one  Who was warm and human  Unwillingly exposed I have been  Neglected in the lonely shore of my illness  Fear of death is rough and arcane  But below these imprisoning machines  My heart beats, my mind thinks  And my soul still feels. | **Tension:**   - Technology vs Humanity: imprisonment underneath the technology of the ICU, craving the contact of “that one who was warm and human” - Life vs Death: Prolonging life at the cost of dignity and respect |
| 2013 | Gristina  Italy  Senior Intensivist reflecting on events 20 years ago | *Grief and renewal: a clinician’s journey* | “It happened almost 20 years ago, in the small hours before dawn.”  “I was overwhelmed by a suffocating burden of the responsibility and shame for things that had not been done, for things that had not been said, for the tragic series of inexplicable forces that had dashed this family to pieces in the space of 4 days.”  ” And the next day, for the ﬁrst time, I went to the funeral of a patient I had treated in the ICU, but the heaviness that had taken hold of me, body and soul, refused to loosen its grip”  “The shield that had allowed me to maintain a safe distance from the suffering around me was shattered, and I felt the sobering but no longer suffocating weight of responsibility that my profession conferred on me.”  As physicians, we are inevitably forced to scramble back and forth between these two poles above all, to be wary of the cold … not only that of the shaded cliffs. | **Tension:**   - Life vs Death: the unfair, seemingly preventable death of a child that should have been full of life; the struggle to keep the boy alive after cardiac arrest - Choice vs Chance: feelings of guilt/regret at not having acted sooner, as if earlier action may have prevented death - Presence vs Absence: Need to look ahead, anticipate events, and act rapidly vs grounding oneself in the present and experiencing life for what it is; not losing oneself in memories of the past |
| 2013 | Do Pico  Argentina  Senior intensivist memoir, reflecting of his professional journey | *Necochea, my personal Ithaca* | I found my fulfillment as intensivist, as teacher, as researcher, but mostly as a man. | **Tension:**   - Technology vs Humanity: infrastructure getting in the way of building an ICU and the human factor of the team overcoming this adversity - Practice vs Prose: Using poetry/literature to contrast with the objectivity of practice |
| 2013 | Bellamo  Australia & New Zealand  Senior Intensivist  Memoir, reflecting on his professional life | *The avoidable death of a boy and the relentless pursuit for evidence* | I remember it like yesterday, but it was 25 years ago.  More and more I believe that compassion and care matter as much as, if not more than, our often deluded beliefs in concepts such as efficacy, physiological gain, and biological manipulation.  I feel it with an unending and unchanged passion within my heart, even as I age and my physical energy is diminished. | **Tension:**   - Choice vs Chance: the choices of certain clinicians ”bad medicine” led to the avoidable death of a boy - Beneficence vs Non-Maleficence: the drive to practice safely (not cause avoidable harm) vs choosing more ”efficacious” treatment - Perfection vs Mediocrity: relentless pursuit of excellence in an effort to prevent harm from sub-optimal medical care |
| 2013 | Gorrea  Uruguay  Senior Intensivist | *Under the red horn of the moon* | It was hard to rationalize the day’s events under the red horn of the moon. I wanted to add thoughts to the feelings that raged through my mind but it was difficult. The vulnerability of the human condition had been unexpectedly revealed in the middle of nowhere and my own vulnerability as an expert intensivist outside of the ICU borders was clearly exposed.  Suddenly, the sky was broken by the red horn of the moon emerging from a cloudy horizon in the middle of my solitude. A crescent moon announcing what poets and mystics have claimed for centuries: life goes on no matter how many tragedies you have to endure.  And then, as it so frequently happens in life, everything changed in a matter of seconds. I had a short moment of indecision that seemed very long. | **Tension:**   - Life vs Death: the vulnerability of life and how chance encounters can change it, affect it, or end it - Control vs Helplessness: the pride/vanity that can come while feeling in control of an ICU team and how that disappears when the author is alone at the side of a road dealing with a disaster response - Ordinary vs Extraordinary: the chaotic, surreal experience of the disaster response contrasted against the serenity of the scene after it had been cleaned up |
| 2013 | Pinsky  USA  Senior Intensivist reflecting on his professional life | *On being an intensivist* | What is it that motivates those who practice acute care medicine to want to do so in the intensive care unit, a place most other doctors and nurses tend to avoid out of fear and trepidation? Is it the feeling of fulfillment when one can be a significant part of one human’s life during a very delicate balance between life and death?  And yet, sometimes, often times, it is not practical medical acumen that serves my patients well, but rather a pure and simple compassion for their mental and physical state and how it impacts those they loved. We are often reminded that death does not only hurt those going through the dying process, but lingers in their surviving loved ones.  …wanderers in the wilderness of disease hoping to find a path to peace that if it could not mean recovery at least would not mean pain and distress. | **Tension:**   - Life vs Death: treading the boundaries between life and death to provide human connection/fulfillment; never prolonging life, just improving the quality of it - Technology vs Humanity: contrasting clinical acumen and objective assessment with human connection/compassion |
| 2013 | MacLaren  Singapore  Intensivist as a father of an ICU patient | *A fresh perfection* | I had cared for hundreds of patients like this in the past but there was something different about this one.  I froze as a sudden unwelcome surge of panic welled up inside of me and shattered my line of thought like a hammer hitting glass. She was my daughter.  I’d never glimpsed the depths of the doctor–patient relationship from the other side. I had not, could not have, appreciated how exposed and vulnerable it makes you feel.  Those that looked after her as gently as if she was their own daughter won my trust and respect. Trust… Hospital buildings might have all the aesthetic appeal of a munitions assembly line but the people who worked in them could and did transcend their cold spirit. | **Tension:**   - Role as Patient vs Physician: contrasting roles as caregiver for ICU patient vs ICU Staff Physician - Technology vs Humanity: comparison of the cold façade of the buildings to the warmth of the human interaction inside them |
| 2013 | Jones  UK  Nurse presenting a relative’s letter | *Farewell to a beloved grandfather* | “the consultant … told us that you was dying and we all decided it would only be fair to you if we let you go”  “I’ve got so many precious memories of you and will keep them with me forever” | **Tension:**   - Life vs Death: humanity and necessity of death contrasted with continued life in the memory of others |
| 2013 | Ranzani,  Brazil  Intensivist  Poem from a patient perspective | *Blindness: perceptions under mechanical ventilation* | Life is something  Wild, Touching your ears, While your mind tries to puzzle out the honest truth. | **Tension:**   - Life vs Death: life springing out after near death experience in ICU - Technology vs Humanity: giving voice to the patient’s experience of mechanical ventilation despite the inability to communicate while vented |
| 2013 | Wang  USA  Intensivist | *A piece of my soul* | …as ICU physicians, our sense of responsibility to do the right thing will likely wane with time as a result of the unrepaired wounds we sustained in our younger days.    …the brief satisfaction of preventing futile care and the associated optimization of ICU resources may simply not be worth the turmoil to our souls. | **Tension:**   - Beneficence vs Non-Maleficence: responsibility to providing care feeling tarnished by facilitating life through violence to the body - Individual vs The System: feelings of the individual drowned by the power of the system |
| 2013 | Bakker  The Netherlands  Intensivist; Non fiction essay; Reflecting on past experience as trainee | *Memorable patients: I’ll be dead on Friday* | It has been long since but the memories are still vivid.  Two patients showed me their capability to predict time of death without any knowledge of complicated scoring systems or many years of clinical experience.  In my current profession as an intensivist I’m sometimes assured I have postponed death using sophisticated technology and treatment schedules. Remembering these two patients always makes me wonder what my contribution really is. | **Tension:**   - Life vs Death: struggle to preserve life against inevitability of death - Choice vs Chance: illusion of control over time of death, humility and recognition of own limits despite the medical decisions made - Permanence vs Impermanence: fleeting moments of patient interaction contrasted against everlasting, vivid memories |
| 2013 | Hernandez  Chile  Senior intensivist reflecting on his professional life and mentorship | *Everything started on a rainy day in Santiago* | We were all paralyzed and moved, and stopped resuscitation minutes thereafter. Much later I understood that it was a matter of respect, of compassion, of dignity.  I had experienced a crucial life event, a sort of epiphany. I had encountered the fascinating alchemy between physiology and humanism  “As Antoine de Sait-Exupery wrote, ‘What makes the desert beautiful is that somewhere it hides a well’” | **Tension:**   - Technology vs Humanity: contrast of published papers, textbooks, and journal articles with the human resilience of navigating adversity, sometimes through the use of literature and music - Hope vs Despair: maintaining hope despite facing multiple challenges |
| 2013 | Hernandez  Chile  Senior Intensivist Poem from the perspective of a dying patient | *The winter steppe (fragments)* | let me find death  Far, far away  Into the heart of the mountains  the mist of death is abrupt or indolent?  who are we?  Just a signal flaring on the monitor screen?  Or simply a chest expanded under the rhythm of the  ventilator?  is not death what I fear  But the abyss of oblivion | **Tension:**   - Life vs Death: prolonging life against the inevitability of death; the ”release” that death provides an anguished life - Technology vs Humanity: technology as a barrier to human connection - Beneficence vs Non-Maleficence: keeping the body alive at the cost of dignity, causation of pain |
